# Involving ornithologists in the surveillance of vancomycin-resistant enterococci.

**DOI:** 10.3201/eid0601.000119

**Published:** 2000

**Authors:** M Sellin, H Palmgren, T Broman, S Bergström, B Olsen


**Pablo Yagupsky and Ron Dagan**
Soroka Medical Center, Ben-Gurion University of the Negev, Beer-Sheva, Israel

### References

1. Graham DR, Band JD, Thornsberry C, Hollis DG, Weaver RE. Infections caused by *Moraxella*, *Moraxella urethralis, Moraxella*-like groups M-5 and M-6, and *Kingella kingae* in the United States, 1953-1980. Rev Infect Dis 1990;12:423-31.
2. deGroot R, Glover D, Clausen C, Smith AL, Wilson CB. Bone and joint infections caused by *Kingella kingae*: six cases and review of the literature. Rev Infect Dis 1988;10:998-1004.
3. Goutzmanis JJ, Gonis G, Gilbert GL. *Kingella kingae* infection in children: ten cases and review of the literature. Pediatr Infect Dis 1991;10:677-83.
4. Yagupsky P, Dagan R, Howard CB, Einhorn M, Kassis I, Simu A. High prevalence of *Kingella kingae* in joint fluid from children with septic arthritis revealed by the BACTEC blood culture system. J Clin Microbiol 1992;30:1278-81.
5. Birgisson H, Steingrimsson O, Gudnasson T. *Kingella kingae* infections in paediatric patients; five cases of septic arthritis, osteomyelitis and bacteraemia. Scand J Infect Dis 1997;29:495-8.
6. Lundy DW, Kehl DK. Increasing prevalence of *Kingella kingae* in osteoarticular infections in young children. J Pediatr Orthop 1998;18:262-7.
7. Yagupsky P, Dagan R, Howard CB, Einhorn M, Kassis I, Simu A. Clinical features and epidemiology of invasive *Kingella kingae* infections in southern Israel. Pediatrics 1993;92:800-4.
8. Yagupsky P, Dagan R, Prajgrod F, Merires M. Respiratory carriage of *Kingella kingae* among healthy children. Pediatr Infect Dis J 1995;14:673-8.
9. Amir J, Yagupsky P. Invasive *Kingella kingae* infection associated with stomatitis in children. Pediatr Infect Dis J 1998;17:757-8.
10. Jensen KT, Schonheyder H, Thomsen VF. In-vitro activity of ß-lactam and other antimicrobial agents against *Kingella kingae*. J Antimicrob Chemother 1994;33:635-40.

### Involving Ornithologists in the Surveillance of Vancomycin-Resistant Enterococci

**To the Editor:** Because migratory birds cross national or intercontinental borders, they are possible long-range vectors for human pathogens such as viruses, *Borrelia burgdorferi* sensu lato, and enteropathogenic bacteria with antibiotic resistance or virulence factors (1,2). Enterococci are ubiquitous in humans and animals and have a propensity for uptake and transfer of glycopeptide antibiotic resistance (3); therefore, the emergence of glycopeptide-resistant enterococci (GRE) in humans is a public health concern. Low-level vancomycin resistance (genotype *vanC-1-3*) is intrinsic in enterococcal species (e.g., *Enterococcus gallinarum, E. flavescens,* and *E. casseliflavus*) that may normally occur in the intestinal flora of some birds. However, the finding of high levels of GRE in wild birds suggests acquisition from an environmental source.

In March 1998, we obtained fecal samples while banding 318 northbound migrating gulls in Malmö, southern Sweden. Using a selective culture procedure with enrichment broth (bile esculin azide broth, Acumedia, LabFab, Ljusne, Sweden) containing vancomycin (8 µg/ml) and aztreonam (60 µg/ml), we isolated vancomycin-resistant *E. faecalis* from a black-headed gull (*Larus ridibundus*). High-level glycopeptide resistance (>256 µg/ml) was demonstrated by E-test (AB Biodisc, Solna, Sweden), and a *van*A genotype was found by polymerase chain reaction amplification (4). This survey protocol can also be used to detect medium to low levels of glycopeptide resistance. Using the same procedure in a study of 230 sub-Antarctic birds on Bird Island, South Georgia, in 1996, we found four GRE isolates with *vanC1* genotype (MIC 3-8 µg/ml).

Many species of gulls have moved into urban areas, where they commonly feed on human trash and deposit feces. The black-headed gull with GRE described above was banded as a fledgling in Malmö in 1995. Birds of this population spend the winter mainly in Western Europe (5), where they forage at garbage dumps, sewage outlets, and agricultural areas. This bird may have acquired GRE in such an area. *Van*A genotype *E. faecium* and *E. faecalis* have been found in poultry and pigs in the Netherlands and Denmark, where the vancomycin analog avoparcin has been used as a growth promoter (6). Manure from such farms may be a GRE source accessible to wild birds.

We have previously reported the introduction into Sweden of multidrug-resistant *Salmonella* Typhimurium by migratory birds (7). The present report further emphasizes the possibility of migratory birds as long-range vectors of bacteria potentially associated with human disease. The risk to humans for GRE from migratory birds may seem insignificant compared with such risk from hospitalization or from eating meat products from GRE-colonized animals. However, if the frequency of birds carrying high-level GRE increases and if amplification in a secondary reservoir or spread through polluted water takes place, spread by migratory birds may become a problem. Bacteriologic surveys of birds may provide vital information for assessing the environmental dispersion of GRE from farms and hospitals. In combination with data about migration patterns and reports of banding recoveries from ornithologists, the potential sources of GRE might be deduced.

This work was supported by the Center for Environmental Research, the Medical Faculty of Umeå University, the Swedish Council for Agricultural Research, and the Swedish Society of Medicine.

**M. Sellin,* H. Palmgren,* T. Broman,* S. Bergström,* and B. Olsen*†**
*Umeå University, Umeå, Sweden; and †Kalmar County Hospital, Kalmar, Sweden

### References

1. Coulson JC, Butterfield J, Thomas C. The herring gull *Larus argentatus* as a likely transmitting agent of *Salmonella montevideo* to sheep and cattle. Journal of Hygiene **London** 1983;91:437-43.
2. Olsen B, Jaenson TGT, Bergström S. Prevalence of *Borrelia burgdorferi* sensu lato infected ticks on migrating birds. Appl Environ Microbiol 1995;61:3082-7.
3. French GL. Enterococci and vancomycin resistance. Clin Infect Dis 1998;27:75-83.
4. Dutka-Malen S, Evers S, Courvalin P. Detection of glycopeptide resistance genotypes and identification to the species level of clinically relevant enterococci by PCR. J Clin Microbiol 1995;33:24-7.
5. Bengtsson K. Migratory routes and wintering areas of Swedish black-headed gull (*Larus ridibundus*) populations. Ornis Svecica 1996;6:17-38.
6. Aarestrup FM, Ahrens P, Madsen M, Pallesen LV, Poulsen RL, Westh H. Glycopeptide susceptibility among Danish *Enterococcus faecium* and *Enterococcus faecalis* isolates of animal and human origin and PCR identification of genes within the VanA cluster. Antimicrob Agents Chemother 1996;40:1938-40.
7. Palmgren H, Sellin M, Bergström S, Olsen B. Enteropathogenic bacteria in migrating birds arriving in Sweden. Scand J Infect Dis 1997;29:565-8.

